# Diversity in orthopaedic surgery residency applications–Where are we?

**DOI:** 10.1371/journal.pone.0355417

**Published:** 2026-09-15

**Authors:** Ameen Chaudry, Rishi Trikha, Thomas Olson, Samuel Clarkson, Chad Ishmael, Kristofer Jones, Alexandra Stavrakis, Rachel Thompson, Nicholas Bernthal

**Affiliations:** UCLA Department of Orthopaedic Surgery, David Geffen School of Medicine at University of California Los Angeles, University of California, Los Angeles, California, United States of America; AIIMS: All India Institute of Medical Sciences, INDIA

## Abstract

**Introduction:**

Gender and racial diversity are key topics for discussion in medical education and, in particular, orthopaedic surgery. Studies have shown patient-physician concordance leads to improved healthcare outcomes. Despite this, the orthopaedic surgery workforce is amongst the least diverse. This study examined trends in gender and race amongst applicants to United States (U.S.) orthopaedic surgery programs to understand the future trajectory of the orthopaedic workforce. With an increasing emphasis on diversity, we hypothesize that gender and racial diversity among orthopaedic surgery applicants have increased.

**Methods:**

Publicly available demographic data from the Electronic Residency Application Service (ERAS) for applicants to surgical residency programs between 2007 and 2019 were collected. Linear regression analysis by residency application year was performed for orthopaedic surgery and other surgical specialties. Z-tests were used to compare respective proportions.

**Results:**

Over the 12-year study period, 18,542 applicants applied to U.S. orthopaedic programs. Of those, 14.9% were female and 28.2% were Black, Indigenous, or People of Color (BIPOC). The percentage of female applicants increased from 11.8% to 19.3% at a rate of 0.41% per year (R = 0.79). The percentage of BIPOC applicants between 2007–2019 ranged from 24.8 to 34.1% annually, trending upwards by 0.44% per year. Compared to other surgical specialties, orthopaedic surgery consistently demonstrated a paucity of female and BIPOC applicants. While African American and Hispanic representation in the orthopaedic surgery applicant pool were comparable to other surgical specialties (6.6% and 8.0%, respectively), Asian applicants were underrepresented at 15.4% compared to 19.3–22.6% for other specialties (p < 0.001).

**Conclusions:**

Application to orthopaedic training programs by female and BIPOC individuals is persistently lower than in other specialties. Despite efforts to increase diversity, diversification has not accelerated signficantly. It remains important for orthopaedic residencies to continue institutional programs and grassroots efforts to encourage a diverse applicant pool.

## Introduction

Diversity in gender and race among physicians is integral to addressing inequalities in healthcare. Diversifying the physician workforce has repeatedly been shown to enhance patient outcomes, foster innovation and improve access [1–3]. Studies have associated surgeon-patient racial concordance with improved postoperative outcomes [4]. Other literature has shown diverse physician populations may be better able to recruit diverse patients into clinical trials, advocate for the needs of diverse populations, and facilitate greater patient satisfaction [5–7]. Despite this, there persists significant gender and racial disparities in most medical fields [8–10]. Orthopaedic surgery is one of the least diverse surgical specialties at the faculty level [11,12].

The percentage of women entering medical school between 1970 and 2001 increased from 11.1% to 47.8%, but female representation at the resident and faculty level in orthopaedic surgery has not matched this pace [12–19]. Recent literature has placed annual female representation amongst orthopaedic surgery residents between just 14.4% and 18% [11–13]. Similarly, only 17.9% of faculty amongst orthopaedic surgery programs and 6.5% of all practicing orthopaedic surgeons are women [12,18,20–22]. Furthermore, the annual percentage of orthopaedic residents who are Black, Indigenous, or People of Color (BIPOC) has ranged between 20.4% and 33.3%, with a negative trend between 2006 and 2015. Notably, BIPOC surgeons comprise just 6% of orthopaedic surgery faculty [12,20,23,24]. At both the resident and faculty level, the rate of gender and racial diversification in orthopaedic surgery has been surpassed by most other surgical specialties [12,20].

Although the gender and racial composition of orthopaedic surgery faculty has been previously described, it is important to assess trends amongst applicants to orthopaedic surgery residency. Analyzing trends amongst residency applicants will elucidate whether the discrepancies in gender and race seen at the resident and faculty levels are congruent with trends amongst applicants. Further, these numbers are reflective of the future composition of the orthopaedic surgery workforce. In projecting the prospective makeup of orthopaedic staff, we will elucidate our trajectory and provide an opportunity to consciously decide whether current efforts are sufficient to achieve our shared goals.

In order to gauge how orthopaedic surgery compares to other surgical specialties in terms of diverse applicant recruitment, we aim to analyze demographic trends amongst surgical residency applicants, a surrogate for projected workforce diversification. Given various known and unknown impacts of the COVID-19 pandemic on residency applicant diversity, combined with literature suggesting female and BIPOC applicants were less likely to apply to competitive surgical specialties as a result of the pandemic, we sought to examine the proportion of female and BIPOC applicants to orthopaedic surgery between 2007−2019, prior to the COVID-19 epidemic [25–27]. Understanding this will provide an objective assessment of our success in recruiting diverse orthopaedic surgery residents, and ultimately a more diverse workforce equipped to provide better patient care. Given a recent emphasis on inclusivity and campaigns fostering diversity in orthopaedics such as the Nth Dimensions Program and the Perry Initiative, we hypothesize that the proportion of female and BIPOC applicants to orthopaedic surgery residency programs has increased in recent years [28,29].

## Materials and methods

Publicly available applicant demographic data including gender, race and ethnicity was collected from the Association of American Medical Colleges’ (AAMC) Electronic Residency Application Service (ERAS) from 2007 to 2019. For this study, applicants were defined as anyone submitting an ERAS application, including U.S. medical school graduates (MD and DO) and international medical school graduates (IMGs). Racial and demographic data was not able to be further stratified by school type (MD, DO, or IMG) from the publicly available ERAS data. Data from 2020 onwards was not publicly available and was excluded to eliminate known and unknown demographic shifts due to the impact of the COVID-19 pandemic on the residency application cycle, as these shifts may not be representative of the true progress made in diversifying the orthopaedic surgery applicant pool.

Data from orthopaedic surgery, general surgery, neurosurgery, otolaryngology, plastic surgery, urology, and vascular surgery applicants were included in this study. On the ERAS application, applicants could identify as either “male” or “female.” Applicants could further identify as American Indian or Alaska Native; Black or African American; Hispanic, Latino, or of Spanish Origin; Native Hawaiian or Other Pacific Islander; Other Race/Ethnicity; Unknown Race/Ethnicity; White; or decline to answer. For our analysis of applicant race, we excluded the small number of “unknown” and “other” responses to focus the analysis on applicants with known racial backgrounds. Applicants who declined to identify a race were still included in the analysis to avoid the critical error of over-representing applicants from under-represented racial groups. In fact, studies have demonstrated that traditionally overrepresented racial groups may perceive a negative bias when applying, potentially affecting their willingness to answer race questions [30,31].

We defined BIPOC applicants as anyone other than those who identifying as White, or those declining to answer. Of note, prior to 2014, the options for “race” did not include those of Hispanic origin. Instead, “Hispanic origin” was indicated using an additional “ethnicity” question, where applicants could choose whether they were of Hispanic origin or not, in addition to self-identifying a “race”. Beginning in 2014, “Hispanic origin” was added as an option for race, and the additional Hispanic ethnicity question was eliminated. As such, prior to 2014, the number of Hispanic applicants was determined from the separate Hispanic ethnicity question.

Linear regression models for the gender and racial distributions of U.S. orthopaedic surgery residency applicants from 2007 to 2019 were then constructed and displayed with corresponding correlation coefficients. Additional regression models were constructed to display trends amongst female and BIPOC applicants to U.S. orthopaedic surgery residency programs as compared to other surgical specialties from 2015 to 2019, as these were the only years in which comprehensive data for all surgical specialties was available. Z-tests were used to compare gender and racial diversity amongst applicants to orthopaedic surgery to the diversity amongst all graduating medical students, and residents and faculty in orthopaedic surgery programs. Z-tests were further used to compare the gender and racial diversity of orthopaedic surgery applicants to that of other surgical applicants. We utilized the Bonferroni Correction for multiplicity to calculate our alpha, defined as 0.05/number of comparisons. Where applicable, a 95% confidence interval was included and abbreviated in the text as “95% C.I.”

## Ethics statement

This study analyzed publicly available, de-identified data and as such did not involve research subjects or require IRB approval.

## Results

Between 2007 and 2019, 18,542 individuals applied to U.S. orthopaedic surgery residency programs, ranging from 1,309–1,582 applicants per year. Of those, 2,757 (14.9%, 95% C.I. 14.6–15.1%) identified as female and 5,226 (28.2%, 95% C.I. 27.9–28.5%) identified as BIPOC.

**Fig 1:**
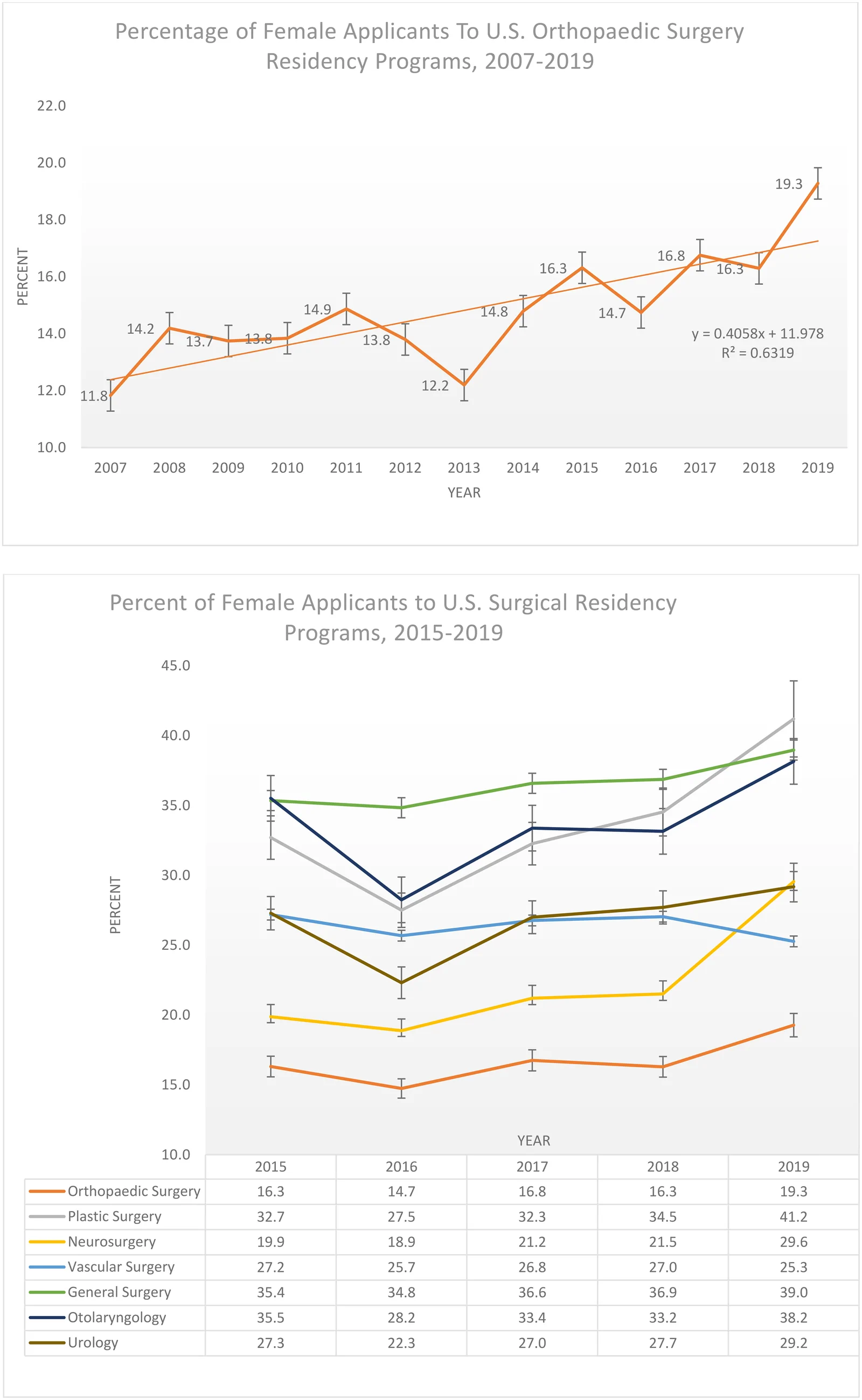
(top) Percent of female applicants to U.S. orthopaedic surgery residency programs (2007 2019). (bottom) Percent of female U.S. surgical residency applicants by specialty (2015–2019). Please note error bars represent standard error.

The number of female applicants ranged from 160 to 304 per year compared to 1,108–1,348 male applicants (Table 1). The percentage of female applicants increased from 11.8% to 19.3% between 2007 and 2019 – a 0.41% increase per year (R = 0.79). This represents an increase of 63.6% (Fig 1). Between 2015 and 2019, the percentage of female applicants to orthopaedic surgery residency (16.8%, 95% C.I. 16.3–17.2%) was significantly lower than the percentage of graduating medical students who were women (46.2%) (p < 0.001). Among surgical specialties, orthopaedic surgery had the lowest percentage of female applicants between 2015 and 2019 (Fig 1). Female representation was significantly lower in orthopaedic surgery than general surgery (36.5%, p < 0.001), otolaryngology (33.7%, p < 0.001), plastic surgery (33.0%, p < 0.001), urology (26.7%, p < 0.001), vascular surgery (26.4%, p < 0.001), and neurosurgery (21.7%, p < 0.001). Of note, in accordance with the Bonferroni Correction for multiplicity, significance was defined as p < 0.05/21 = 0.0024 whenever comparing the percentage of applicants between the seven surgical specialties studied.

**Table 1:** Number of female, BIPOC, and total applicants to U.S. orthopaedic surgery residency programs (2007–2019).

Year	Female	BIPOC	Total
**2007**	168	523	1420
**2008**	193	485	1360
**2009**	188	511	1368
**2010**	213	566	1539
**2011**	226	547	1520
**2012**	189	437	1370
**2013**	160	452	1312
**2014**	234	586	1582
**2015**	216	455	1324
**2016**	193	388	1309
**2017**	247	470	1474
**2018**	226	444	1387
**2019**	304	544	1577

From 2007 to 2019, between 302 and 507 BIPOC graduating medical students applied to orthopaedic surgery residency each year, accounting for 24.8% to 34.1% of applicants each year (excluding those who did not identify a race; Table 1).

**Fig 2:**
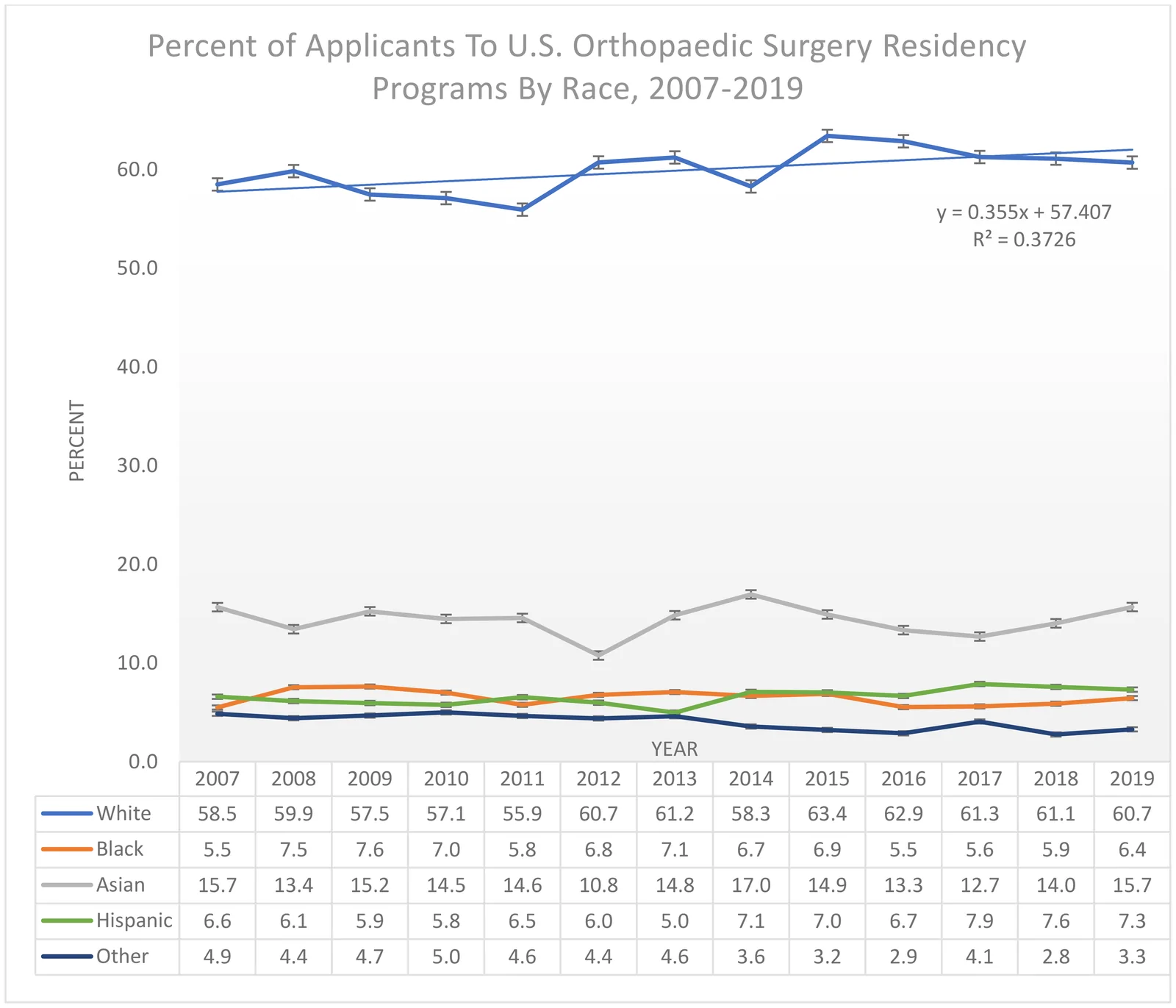
Percent of applicants to U.S. orthopaedic surgery residency programs by race (2007 – 2019). Between one and seven Native Hawaiian/Pacific Islander, and six to fifteen American Indian/Alaskan Native applicants applied annually (not shown). Please note error bars represent standard error.

The contribution of BIPOC students to the applicant pool increased by 0.44% per year between 2007 and 2019 (R = 0.68). During this time, there were 11,785 White applicants (95% C.I. 63.2–63.9%), ranging between 830 and 980 applicants per year (65.9% to 75.2%). Asian applicants were the second most represented racial group in the applicant pool, with 2,839 applicants between 2007 and 2019 (95% C.I. 15.0–15.6%). There were between 162 and 285 Asian applicants each year (13.3% to 19.4%). Hispanic applicants were the next most represented racial group with 1,297 applicants in this period (95% C.I. 6.8–7.2%), and between 69 and 119 applicants each year (5.6% to 8.7%). Thereafter, African Americans made up the next largest group of applicants, with 1,279 applicants in this time period (95% C.I. 6.7–7.1%) and between 73 and 119 applicants each year (6.0% to 9.2%). There were between one and seven Native Hawaiian/Pacific Islander, and between six and fifteen American Indian/Alaskan Native applicants annually. Notably, between 38 and 85 applicants each year chose “Other” as their race, and between 45 and 194 applicants each year did not identify a race (Fig 2).

**Fig 3:**
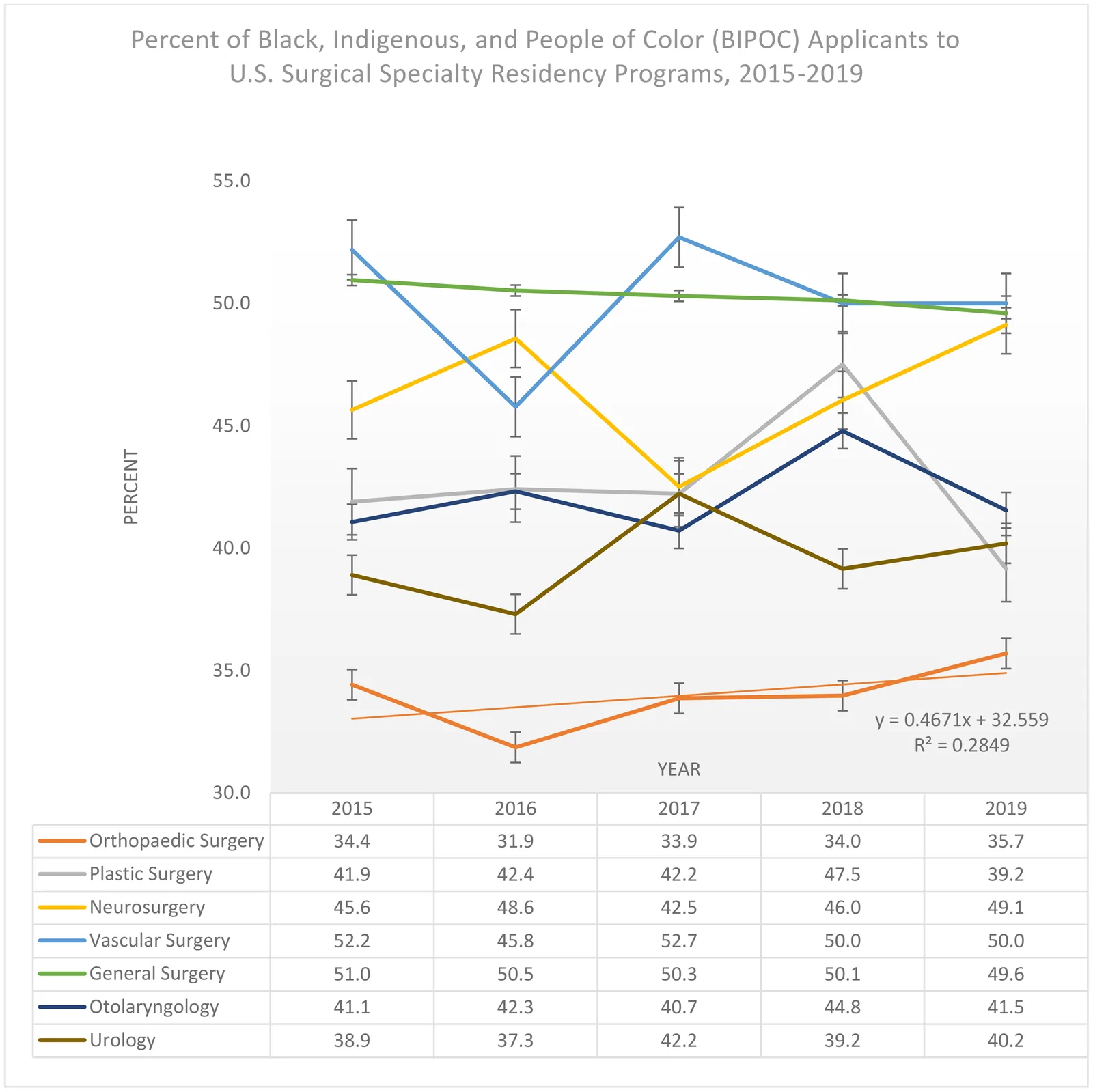
Percent of Black, Indigenous, or People of Color (BIPOC) U.S. surgical residency applicants by specialty (2015 – 2019). Please note error bars represent standard error.

From 2015 and 2019, the percentage of BIPOC orthopaedic surgery applicants (32.6%, 95% C.I. 32.0–33.2%) was significantly lower than the percentage of graduating medical students who were BIPOC (55.4%, p < 0.001).Similarly, orthopaedic surgery had the lowest percentage of BIPOC applicants when compared to other surgical specialties. This difference was statistically significant in comparisons with all other included surgical specialties: vascular surgery (58.0%, p < 0.001), general surgery (57.3%, p < 0.001), neurosurgery (55.3%, p < 0.001), plastic surgery (45.2%, p < 0.001), otolaryngology (42.8%, p < 0.001), and urology (42.0%, p < 0.001) (Fig 3).

**Fig 4:**
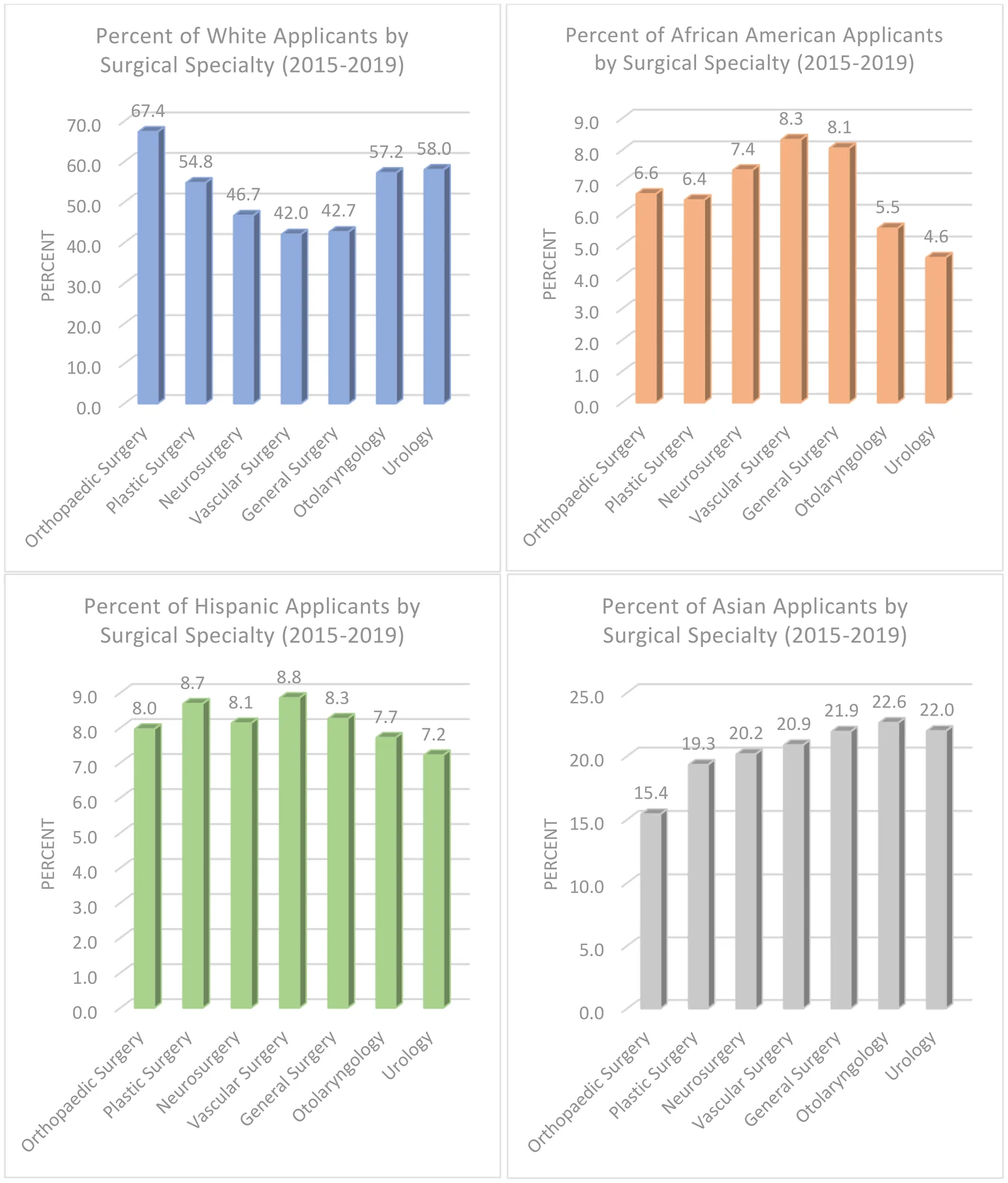
Percent of applicants to U.S. surgical specialty residency programs by race (2015 – 2019).

The percentage of African American applicants to orthopaedic surgery between 2015 and 2019 (6.6%, 95% C.I. 6.3–6.9%) was significantly lower than the percentage of graduating African American medical students (7.2%, p < 0.05) and decreased by 0.17% per year between 2007 and 2019 (R = 0.63). However, the percentage of African American orthopaedic surgery applicants was significantly greater than the percentage of African American applicants to urology (4.6%, p < 0.001); not significantly different than the percentage who applied to otolaryngology (5.5%, p < 0.05), neurosurgery (7.4%), plastic surgery (6.4%), or vascular surgery (8.3%, p < 0.01); and significantly less than the percentage that applied to general surgery (8.1%, p < 0.001) (Fig 4).

Similarly, the percentage of Asian applicants to orthopaedic surgery residency between 2015 and 2019 (15.4%, 95% C.I. 15.0–15.8%) was significantly lower than the percentage of graduating Asian medical students (23.7%, p < 0.001) and decreased by 0.23% per year between 2007 and 2019 (R = 0.46). Moreover, the percentage of Asian applicants to orthopaedic surgery was significantly lower than the percentage to urology (22.0%, p < 0.001), general surgery (21.9%, p < 0.001), otolaryngology (22.6%, p < 0.001), vascular surgery (20.9%, p < 0.001), neurosurgery (20.2%, p < 0.001), and plastic surgery (19.3%, p < 0.001).

In contrast, the percentage of Hispanic orthopaedic surgery applicants between 2015 and 2019 (8.0%, 95% C.I. 7.7–8.3%) was not significantly different than the percentage of graduating Hispanic medical students (7.9%, p = 0.93) and slightly increased by 0.06% per year between 2007 and 2019 (R = 0.28). The percentage of Hispanic applicants to orthopaedic surgery was not significantly different than the percentage to urology (7.2%, p = 0.25), otolaryngology (7.7%, p = 0.67), neurosurgery (8.1%, p = 0.79), general surgery (8.3%, p = 0.42), plastic surgery (8.7%, p = 0.28), or vascular surgery (8.8%, p = 0.23).

## Discussion

The diversity of residency applicants is a surrogate for future workforce diversification. By utilizing applicant demographic data from various surgical specialties, the current study demonstrates a significantly lower percentage of female and BIPOC applicants to orthopaedic surgery residency compared to other surgical specialties, a concerning indicator for persistent homogeneity in the orthopaedic surgery pipeline. This is concerning as a less diverse workforce may be underequipped to provide quality care to a diverse patient population. This can have profound implications for the patient-physician relationship, being able to advocate for the unique needs of diverse patients, and tailoring the practice of medicine to each patient’s individual needs. A less diverse workforce may also be less culturally competent in managing the complex medical, social, and psychologic components of delivering healthcare for each patient.

While there was a modest increase in female applicants during the included time period, the percentage of African American and Asian applicants decreased. This parallels literature showing that the percentage of BIPOC residents in orthopaedic surgery decreased from 33.29% in 2006 to 22.48% by 2015 [12,32]. One factor likely contributing to decreased diversity in the applicant pool is the availability of mentors of similar backgrounds. Having fewer female and BIPOC mentors likely detracts from a potential applicant’s interest in the field. In fact, women and BIPOC applicants who attend medical school with more female and BIPOC faculty members are more likely to apply into the fields of those faculty members [17,23]. Additionally, departments with more women in faculty and leadership positions tend to have a higher proportion of female residents [17,23]. Moreover, female applicants cite the presence of female and BIPOC residents and faculty as important factors when deciding whether or not to apply to orthopaedic surgery residency [33,34]. However, nearly 20% of orthopaedic surgery programs include exclusively male trainees, and 39.5% of orthopaedic surgery programs have no BIPOC trainees, likely a deterring factor for female and BIPOC applicants [19,35].

While progress has been made toward increasing female representation in orthopaedic surgery, the rate of increase and overall representation still lags behind other surgical specialties. Poon et al. demonstrated a 0.34% annual increase in the percentage of female orthopaedic surgery residents between 2006 (10.98%) and 2015 (14.41%), with recent literature suggesting a continued trend of increasing female representation [11–13]. The percentage of female faculty and residents in orthopaedic surgery during this time is nearly identical to the percentage of female applicants to orthopaedic surgery found in this study [12,18,20–22]. Given this relatively equivalent rate of current female faculty representation and female orthopaedic residency applicants, female representation in orthopaedic surgery is at risk of remaining stagnant.

The match rates of BIPOC and female applicants have also been studied. BIPOC applicants have alarmingly lower match rates than their White counterparts [12,32]. Between 2015–2019, White applicants matched at a rate of 63.4%, which was significantly greater than rates for African Americans (39.5%), Hispanics (54.2%), and Asians (54.1%) [32]. Moreover, female match rates have been similar to their male counterparts in orthopaedic surgery (56.2% vs. 55.2%, respectively) [32]. Thus, current match rates for BIPOC and female applicants will not provide an avenue for diversifying the orthopaedic surgery workforce.

When female medical students do pursue orthopaedic surgery, unfavorable experiences during the interview process may preclude other potential applicants from applying. Despite an increased focus on adherence to interview guidelines, there remains the possibility of inappropriate questioning during the interview process. In 2016, O’Connor et al. found that women faced much higher levels of inappropriate or biased questioning than male applicants [36]. These topics included family planning (61% vs 8%), marital status (24% vs 7%) and children (33% vs 4%). Such questions not only disadvantage applicants who face them, but also add to perceptions of the field being hostile towards women, discouraging other women from applying in the future. While such questions may not be rooted in ill-intent, the implicit bias of the interviewer may ultimately be detrimental to the applicant’s pursual of orthopaedic surgery.

Limitations of this study include that prior to 2014, Hispanic origin was asked in a separate “ethnicity” question, whereas all other racial groups were asked about in a separate “race” question. As a result, individuals of Hispanic origin would list themselves as Hispanic on the ethnicity question, and possibly “other” or “White” on the race question, potentially resulting in a small number of additional responses for those other racial groups.. Given the low variation in Hispanic representation throughout the study period, this likely did not affect the analysis significantly. Further, some individuals identify with multiple races, and thus may have been double-counted for selecting multiple races, but given the large applicant pool, it is unlikely that this significantly altered the racial analysis. Additionally, due to perceptions of bias against certain races, individuals may have chosen to select “unknown” or “other.”

Another limitation is ERAS only allowed individuals to identify as “male,” “female” or “unknown” for gender, necessarily excluding gender non-binary and possibly excluding transgender individuals. Interestingly, only one individual identified as “unknown” from 2015 to 2019. Notably, while this study describes a statistically significant underrepresentation of Asian applicants to orthopaedic surgery compared to other specialties, it cannot be determined from the available data whether certain subgroups (for example South Asian, East Asian, or Middle Eastern) are differentially affected. Finally, it is difficult to describe an expected rate of diversification, and thus diversification in orthopaedic surgery can only be described comparatively against other specialties and the diversity amongst medical school graduates as a whole.

Despite these limitations, this work represents an accurate and much-needed analysis of the diversification trends in orthopaedic surgery application. Although one previous publication sought to answer a similar question, it did not account for those applicants who listed themselves as “unknown” or “other,” and therefore we believe this manuscript provides a more accurate assessment of the trends in orthopaedic surgery application [37].

This analysis suggests that, as a field, orthopaedic surgery may not yet be on a path to achieve its oft-stated goal in enhancing diversity. Although more recent literature has been encouraging, more time is needed to assess diversification trends in the post-COVID era. While specific interventions to correct this course are beyond the scope of this study, this analysis does highlight a persistent lack of diversity at one of the earliest stages of our orthopaedic “pipeline” – residency applicants. We lean on the plethora of work in this field across specialties to identify solutions: orthopaedic surgery programs must continue to make efforts to promote favorable experiences for those of under-represented backgrounds; programs must appropriately promote female and minority faculty into leadership positions; and efforts to recruit diverse applicants must continue during early medical education. While multiple programs have attempted to attract students from under-represented backgrounds to orthopaedic surgery (i.e., the Perry Initiative, which focuses on recruiting women to orthopaedic surgery, and the Nth Dimension program, which engages both women and under-represented groups), it is clear more must be done. These programs have demonstrated that early exposure is an effective recruiting tool and this study suggests we must focus on these early time points [38,39]. Orthopaedic surgery needs to increase access to such recruitment programs if it is to reach its desired targets and recruit more diverse applicants. We must also encourage the appropriate advancement of diverse faculty and mentors who can not only serve as role models for existing female and BIPOC medical students, but also provide examples of culturally competent care and extend outreach to diverse patient populations in hopes of inspiring diverse future physicians. Moreover, it remains important to consider the unique challenges of applicants from diverse backgrounds in their journey toward orthopaedic surgery residency.

Ultimately, diversification efforts over the last decade within orthopaedic surgery may not yet have translated into significant increases in the applicant pool, especially when compared to other surgical specialties. The percentage of female orthopaedic surgery applicants is similar to current resident and faculty numbers, which likely indicates stagnation in diversification efforts. Additionally, Hispanic representation has remained relatively stable while African American and Asian representation among orthopaedic surgery residency applicants has actually decreased. If we are to achieve our stated goals of enhancing diversity within our field, orthopaedic leaders must actively pursue diverse residents and faculty through current initiatives and novel programs aimed toward promoting inclusivity so that we may increase interest in orthopaedic surgery in our youngest learners.
